# Complete genome sequence of *Lactobacillus iners* DSM 13336 determined by Oxford Nanopore Technologies long-read sequencing

**DOI:** 10.1128/mra.00433-26

**Published:** 2026-10-15

**Authors:** Cyrille Jarrin, David Vilanova, Patrick Robe, Joran Dupont, Perrine Lemagnen, Antoine Robin, Amandine Scandolera, Romain Reynaud

**Affiliations:** 1 Givaudan France SAS 396259, Ramonville Saint-Agne, France; 2 Genomictales, Encamp, Andorra; 3 Givaudan France SAS, Pomacle, France; Fluxus Inc., Sunnyvale, California, USA

**Keywords:** genome, long read, vaginal microbiota

## Abstract

*Lactobacillus iners* is a key member of the human vaginal microbiota. We present the complete genome sequence of strain DSM 13336, obtained with Oxford Nanopore Technologies. The obtained single circular chromosome (1,337,293 bp) offers a high-quality genomic resource for *L. iners*, underscoring the distinctive features of the DSM 13336 strain.

## ANNOUNCEMENT

*Lactobacillus iners* is a gram-positive, facultatively anaerobic bacterium that represents a predominant member of the human vaginal microbiota  (1, 2). A large proportion of women of reproductive age harbor a vaginal community dominated by *L. iners * (3, 4). This species contributes to the maintenance of low vaginal pH through lactic acid production but is also frequently associated with microbial profiles characteristic of bacterial vaginosis  (5, 6). Publicly available genome sequences of *L. iners* reference strains remain limited and are typically represented by draft assemblies. For example, the closely related strain DSM 13335 (GCF_000160875.1) comprises 12 scaffolds. To date, only one complete, circularized genome has been reported (7, CP048798.1) for a strain (KY) that is not publicly available. Here, we present the complete genome sequence of *L. iners* DSM 13336, obtained from the Leibniz Institute DSMZ–German Collection of Microorganisms and Cell Cultures (Braunschweig, Germany), providing an enhanced genomic resource for this clinically relevant species.

*L. iners* DSM 13336 was cultured in 200 mL de Man, Rogosa and Sharpe (MRS) broth supplemented with L-cysteine and L-glutamine (MRS-CQ). Incubation was done at 37°C in an anaerobic atmosphere generated using a GasPak EZ Anaerobic Gas Generating reagent (BD). After 23 h, the cells were collected, and the full biomass (1 g) was used for DNA extraction, using NucleoBond Buffer Set III and NucleoBond AXG silica-based anion exchange columns (Macherey-Nagel), following the manufacturer’s instructions. DNA quality and quantity were assessed by spectrophotometry, showing 280/260 and 260/230 ratios of 1.86 and 2.32, respectively, with an estimated DNA concentration of 120 ng/µL in a volume of 600 µL.

Sequencing library was prepared from 1 µg of extracted genomic DNA, without shearing or size selection, using the Oxford Nanopore Technologies SQK-LSK114 ligation kit. The library was then loaded onto a MinION Mk1D with a FLO-MIN114 flow cell (R10.4.1). Sequencing run was controlled with MinKNOW v25.09.17  (8). Base calling was performed in Dorado v7.11.2 (SUP model) (9). The raw output comprised 3,260,254 reads, with an N50 of 5,497 bp, a mean read quality of Q18.8, and an estimated 150×  assembly coverage. Read quality control and trimming were conducted using Chopper v0.12.0 (minimum Q score 15, minimum length 200 bp) (10). *De novo* assembly was performed using Flye v2.9.6  (11) with an estimated genome size of 1.3 Mbp. The assembly was subsequently polished with Medaka v2.2.0 (model r1041_e82_400bps_sup_v5.2.0) (12). Genome quality was evaluated using QUAST v5.3.0  (13), and the average nucleotide identity (ANI) relative to *L. iners* DSM 13335 and other published *L. iners* genomes was calculated using skani v0.3.1  (14). Genome annotation was performed using the NCBI Prokaryotic Genome Annotation Pipeline  (15) during GenBank submission; the annotation statistics are summarized in Table 1. Default parameters were applied, except where otherwise specified.

**TABLE 1:** Sequencing and assembly statistics for *Lactobacillus iners* DSM 13336

Metric	Value
Number of reads	3,260,254
Total genome length (bp)	1,337,293
Read length N50 (bp)	5,497
Assembly coverage	150×
GC content (%)	33.15
Number of predicted genes	1,310
Number of CDS	1,222
Number of tRNA genes	67
Number of rRNA genes	18
ANI vs DSM 13335 (%)	98.86
ANI vs *L. iners* KY (%)	98.34

The final assembly consisted of a single circular chromosome of 1,337,293 bp, with a GC content of 33.15% and an N50 of 1,337,293 bp. Circularity was inferred by Flye from terminal contig overlap and confirmed in the deposited chromosome sequence. No additional reorientation or rotation was applied beyond that performed by the assembler or during deposition. The ANI relative to the reference strain DSM 13335 was 98.86%. Pairwise ANI comparisons with other published *L. iners* genomes (strains KY, C0094A1, and C0254C1; GenBank accessions CP048798.1, CP049223, and CP049231) ranged from  98.34 to 98.61%. Interestingly, Kwak and coworkers  (7) described three putative core genes (*INY*,* ZnuA*, and* hsdR*) of *L. iners*. Among these, only two (*INY *and* ZnuA*) were identified in the present genome, showing 99.36% and 99.67% nucleotide sequence identity, respectively. Together, these results confirm that DSM 13336 represents a distinct strain and that the present genome provides a complete, high-quality genomic resource for *L. iners*.

## Data Availability

The complete genome sequence of *Lactobacillus iners* DSM 13336 has been deposited in GenBank under the chromosome accession CM148630.1 as part of assembly GCA_056120565.1. Raw sequencing reads were deposited in the NCBI Sequence Read Archive under BioProject PRJNA1432907 and were run under accession SRR37488067. The BioSample accession number was SAMN56351716.
